# Data Sharing, Biopsies and Patient Confidentiality in a Precision Medicine Trial for Childhood Cancer: A Mixed Method Study of Parents, Oncologists, and Scientists’ Perspectives

**DOI:** 10.3390/jpm15110531

**Published:** 2025-11-02

**Authors:** Yvanna Lei, Kate Hetherington, Rebecca Daly, Niki Rensen, Brittany C. McGill, David S. Ziegler, Loretta M. S. Lau, Vanessa Tyrrell, Jonathan Karpelowsky, Mark J. Cowley, Katherine M. Tucker, Michelle Haber, Paulette Barahona, Claire E. Wakefield

**Affiliations:** 1 School of Clinical Medicine, Discipline of Pediatrics, UNSW Medicine and Health, UNSW Sydney, Sydney, NSW 2031, Australia; 2 Behavioural Sciences Unit, Kids Cancer Centre, Sydney Children’s Hospital, Randwick, NSW 2031, Australia; 3 Princess Maxima Center, 3584 CS Utrecht, The Netherlands; 4 Kids Cancer Centre, Sydney Children’s Hospital, Randwick, NSW 2031, Australia; 5 Children’s Cancer Institute, UNSW Sydney, Sydney, NSW 2031, Australia; 6 Department of Paediatric Surgery, Children’s Hospital at Westmead, Westmead, NSW 2145, Australia; 7 Children’s Cancer Research Unit, Kids Research Institute, Children’s Hospital at Westmead, Westmead, NSW 2145, Australia; 8 Division of Child and Adolescent Health, University of Sydney, Sydney, NSW 2145, Australia; 9 Kinghorn Centre for Clinical Genomics, Garvan Institute, Darlinghurst, NSW 2010, Australia; 10 Hereditary Cancer Centre, Department of Medical Oncology, Prince of Wales Hospital, Randwick, NSW 2031, Australia; 11 Prince of Wales Clinical School, UNSW Sydney, Randwick, NSW 2031, Australia; 12 Division of Quality of Life and Pediatric Palliative Care, Department of Pediatrics, Stanford University and Stanford Medicine Children’s Health, Palo Alto, CA 94304, USA

**Keywords:** precision medicine, pediatric oncology, scientists, parents, data sharing, biopsy, confidentiality

## Abstract

**Background/Objectives**: Precision medicine is transforming care for children with cancer, but raises new challenges. We explored parents’, oncologists’ and scientists’ perspectives on three aspects of a precision medicine trial for poor prognosis childhood cancer: data sharing, requests for additional tumor biopsies, and confidentiality. **Methods**: Data were collected through PRISM-Impact, a psychosocial sub-study within the Zero Childhood Cancer Program’s PRISM trial. Parents completed questionnaires at enrolment and one year later, and an optional interview after receiving their child’s trial results. Bereaved parents completed a questionnaire six months after bereavement (T1B). Oncologists and scientists were interviewed one year following trial commencement. Quantitative data were analyzed descriptively, and qualitative data thematically. **Results**: Parents (*n* = 126) considered additional tumor biopsies acceptable when risks were low and their child or oncologist supported the request. Oncologists (*n* = 26) emphasized weighing risk–benefit, ensuring parents felt fully informed, and research value. Most parents supported data sharing (≥89–96%), including after bereavement, despite potential privacy concerns. Parents supported overseas and interstate testing, and scientists having access to identifiable health information. Scientists (*n* = 10) found working with identifiable data emotionally challenging. **Conclusions**: Parents, oncologists, and scientists showed high acceptance of procedural aspects of precision medicine. Future trials should address privacy concerns and ensure informed consent recognizes that parents’ high acceptability of procedures may be linked to their hopes for benefit, reinforcing the need for informed consent.

## 1. Introduction

Childhood cancer remains a leading cause of disease-related death amongst children and adolescents worldwide [1,2]. Despite improved survival rates, children with rare, relapsed, or refractory cancers often still have a poor prognosis [3] with new treatment approaches urgently needed [4]. Precision medicine uses comprehensive molecular profiling to understand a patient’s cancer and tailor treatments [5,6], aiming for more effective and less toxic therapies [7]. Several pediatric precision medicine programs are underway [8] requiring parents, oncologists, and scientists to consider procedural factors, such as additional tumor biopsies, sharing patient data, and confidentiality in a clinical research setting.

Participation in precision medicine programs relies on the availability of tissue samples for molecular profiling [9]. Samples from standard procedures are usually sufficient after routine clinical testing [10]; however, if unavailable or poor in quality or quantity, an additional biopsy may be required [9,11,12] along with blood specimens [13]. Parents must weigh the risks of such procedures against potential benefits. Parents have reported that factors such as quality of life, survival, and cost are more influential than their child requiring a biopsy when deciding on trial enrolment [14]. However, some parents hesitate to join future trials if biopsies are required [15,16]. Willingness tends to increase when oncologists recommend the procedure [15], underscoring the need to understand oncologists’ perspectives. Clinicians, including oncologists and surgeons, face challenges weighing up biopsy risks for precision medicine trial enrolment [17,18]. Oncologists are more likely to recommend a biopsy if the likelihood of finding a molecular target is high, invasiveness and morbidity rate are low, and results could influence clinical management [19]. Since molecular analysis may not directly benefit patients [20] and the value of research biopsies remains debated [21], further exploration of oncologists’ perspectives is warranted.

In the context of a pediatric precision medicine research trial, parents may also be asked to consent to share their child’s data. For the purposes of this study, “data” refers to de-identified genetic information (germline and tumor).

Clinical data sharing is essential to advance research output and address knowledge gaps in pediatric oncology [22,23,24]. As childhood cancers are rare and histologically diverse [25], and with precision medicine becoming standard care, large datasets are crucial to maximize benefits [26]. Parents may need to consider the new challenges posed by genomic analysis in precision medicine. Despite efforts to protect privacy during data sharing, risks persist, including the risks to patients’ personal information [27]. Sharing pediatric data is further complicated due to ethical concerns surrounding the use of data from participants who are unable to provide consent themselves (i.e., children < 18 years) [28,29].

There is currently no literature on parents’ attitudes toward data sharing within a precision medicine trial. Existing studies show both adult patients and parents of pediatric patients are largely willing to share genomic data [30,31], but parents generally prioritize privacy more [30]. Parents also expressed concerns about unspecified future risks for their children with evolving technologies or laws [30,31]. Since parents’ willingness to share data can depend on anticipated benefit [32], it would be valuable to understand parents’ attitudes following the return of trial results, including bereaved parents. Precision medicine trials are bringing non-patient-facing professionals, such as scientists, closer to clinical care than ever before. Scientists involved in precision medicine trials have reported benefits such as seeing how their contributions directly influence patient outcomes through exposure to clinical decision-making during molecular tumor board (MTB) meetings [33]. This involvement means scientists now have greater access to identifiable patient information pertaining to the samples they are analyzing. Currently, our understanding of scientists’ experiences with managing confidentiality in this setting is lacking.

Given that the procedural aspects and confidentiality issues described can raise new ethical considerations [34,35,36], it is important to understand parents’, oncologists’, and scientists’ attitudes toward these matters to inform the development of future trials. Therefore, we aimed to answer the following research questions:

1. What are parents’ attitudes toward sharing their child’s anonymous trial data and sending samples interstate and overseas?
2. How do parents and oncologists view the potential need for additional tumor biopsies and blood samples in a pediatric precision medicine trial?
3. What are parents’ and scientists’ perspectives on confidentiality in a pediatric precision medicine trial?

## 2. Materials and Methods

### 2.1. PRISM

The PRecISion Medicine for Children with Cancer (PRISM) trial is embedded in the Zero Childhood Cancer Program (ZERO), Australia’s national precision medicine program for children (≤21 years) with poor prognosis cancer (expected survival <30%; Australian and New Zealand Clinical Trials Registry, NCT03336931, 8 November 2017) [37]. ZERO’s PRISM trial utilizes comprehensive genomic profiling of the tumor, germline, and, where possible, targeted drug screening and/or drug efficacy testing, for identification of potentially more likely effective personalized treatment recommendations [37]. The results of this testing are brought to an MTB consisting of clinicians and scientists, which reviews each patient’s results and delivers treatment recommendations to the patient’s oncologist [33]. PRISM’s information sheet and consent form inform parents that the trial may require their child to undergo an additional tumor biopsy. It also states that PRISM testing is conducted in laboratories both in Australia and overseas, and that de-identified germline and tumor genetic information will also contribute to research databases or registries in Australia or overseas. All staff in the ZERO program are required to undertake patient privacy training.

### 2.2. PRISM- Impact

**Figure 1:**
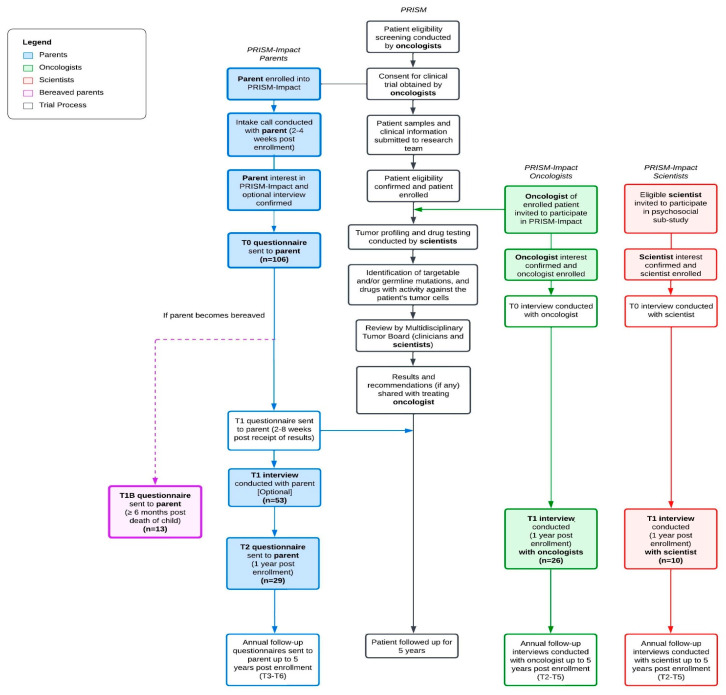
PRISM and PRISM-Impact study design and procedures in parallel. Note: Words in bold indicate the participant groups relevant to this study. Boxes shaded indicate the procedures and timepoints relevant to this study.

PRISM-Impact is a psychosocial substudy that runs alongside ZERO’s PRISM trial (Figure 1). Parents opted in to contact from the PRISM-Impact team through the PRISM trial consent form. PRISM and PRISM-Impact were conducted in accordance with the Declaration of Helsinki and received institutional board approval of Hunter New England Local Health District (ethical and governance approval number: HREC/17/HNE/29, 2 May 2017). PRISM-Impact uses a prospective mixed-methods design to explore families’, healthcare professionals’, and scientists’ experiences of participating in PRISM. The current study presents a subset of data collected as part of PRISM-Impact (between September 2017 and September 2021). Due to different consent processes with adult patients, we present data only from parents of patients aged 0–17 years. PRISM is now closed to recruitment, but an ongoing follow-up of several remaining PRISM-Impact families is still underway.

### 2.3. Procedure

Shortly after enrolling in PRISM, we contacted parents to confirm their consent to participate in PRISM-Impact. We sent parents questionnaires at enrolment (T0) and one year later (T2). We invited bereaved parents to complete an adapted questionnaire six months after their child’s death (T1B). A subset of parents also took part in an optional semi-structured interview after receiving PRISM results (T1). We invited oncologists and scientists involved in the trial to participate in a semi-structured interview one year following trial commencement (T1) via a personalized email from the PRISM investigator (DZ). Interview topics varied by professional group and aligned with each participant’s expertise. We collected clinical information (e.g., diagnosis) from the PRISM study central database. The PRISM-Impact questionnaires and interviews included validated measures (adapted from refs. [15,19,38]) and open-ended interview questions with probes to elicit detailed responses. The questionnaires and interviews were developed and pilot tested with input from a multidisciplinary expert panel. We added measures assessing parents’ attitudes towards data sharing into the PRISM-Impact study via an ethics amendment approved on 1 April 2019, and so were completed by only a subset of the PRISM-Impact sample. Supplementary Table S1 summarizes the parent questionnaires and semi-structured interviews, which cover topics including demographics, views on data sharing, and perspectives on additional biopsies. Seven psychosocial researchers (JY, FL, EB, CR, JH, RD, and NH) conducted in-depth semi-structured interviews, which lasted 28.35 min on average (range: 15–76 min). Interviews were audio-recorded and transcribed verbatim with identifiable information (e.g., participant names) removed prior to analysis.

### 2.4. Data Analysis

We used a convergent parallel design for this mixed-method study [39]. We analyzed quantitative and qualitative data independently and integrated findings afterwards.

### 2.5. Quantitative Analyses

We used the Statistical Package for the Social Sciences (v28.0) to perform quantitative analyses [40]. We used descriptive statistics to report on participants’ demographics, parents’ attitudes toward data sharing according to recipient and purpose, and parents’ perceptions of the benefits and negatives of data sharing. We collapsed the latter responses into three categories (positive = ‘benefits outweighing negatives’, negative = ‘negatives outweighing benefits’, neutral = ‘benefits and negatives are equal’) to simplify the presentation and interpretation and descriptively examined each parent’s change in response between enrolment (T0) and one year post enrolment (T2) and change in perceptions from enrolment (T0) to post bereavement (T1B).

### 2.6. Qualitative Analyses

We used a thematic analysis with an inductive approach [41] with the support of coding software NVivo [42] to analyze qualitative data. One researcher (Y.L.) became familiar with the transcripts and developed an initial coding framework guided by the study aims [41]. The initial coding framework was discussed and revised (Y.L. and K.H.) to reach consensus. Interviews were then coded line by line (Y.L.) to develop the themes and subthemes, which were critically appraised via ongoing discussion (Y.L and K.H). We then aligned these themes with the quantitative dataset and extracted illustrative quotes to create a cohesive overview.

## 3. Results

### 3.1. Participants

#### 3.1.1. Parents

Data sharing measures were completed by 106 parents at T0, 29 parents at T2, and 13 parents at T1B. In total, we included data of 126 parents from 110 families in this manuscript (see supplementary material note; for information on factors associated with participation and attrition). During intake, 63 parents opted into a T1 interview, and 53 parents completed the interview.

Supplementary Table S2 summarizes parents’ baseline characteristics (*n* = 126, 61.1% mothers). Table 1 summarizes their children’s (*n* = 110) characteristics which included an equal number of girls (50%) and boys, had an average age of 7.8 (±5.5) years at cancer diagnosis, and 8.9 (±5.6) years at PRISM enrolment. Like the overall PRISM trial cohort, patients had been diagnosed with a central nervous system tumor (36.4%), sarcoma (31.8%), hematologic malignancy (16.4%), neuroblastoma (7.3%), or other tumor (8.2%). Around half (50.9%) had experienced at least one relapse prior to enrolment in PRISM.

#### 3.1.2. Oncologists and Scientists

We invited 57 oncologists to participate, 30 of whom opted in (response rate 52.6%) and 26 completed interviews (participation rate 86.6%). We invited 24 scientists to participate, 11 of whom opted in (response rate 45%) and 10 completed interviews (participation rate 90.9%). Table 2 summarizes the characteristics of participating oncologists and scientists.

### 3.2. Research Question 1: Attitudes Toward Sharing of the Child’s Anonymous, Individual Trial Data

**Figure 2:**
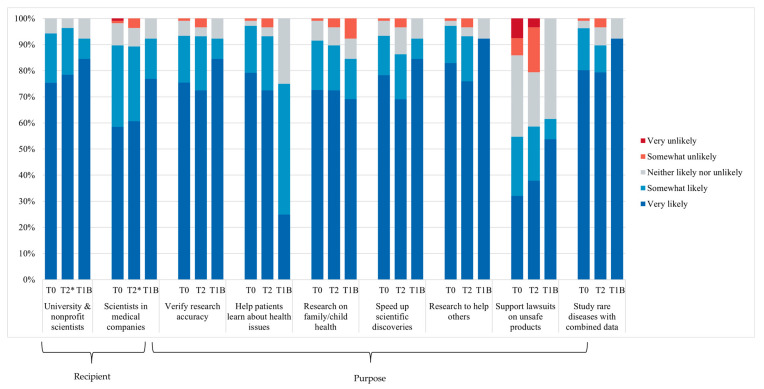
Parents’ attitudes toward data sharing, according to recipient and purpose at T0 (*n* = 106), T2 (*n* = 29), and T1B (*n* = 13). T2* missing data *n* = 1.

Figure 2 summarizes parents’ attitudes toward data sharing at each time point (T0, T2, and T1B). Across all time points, most parents (≥89–96%) reported that they were very or somewhat likely to share their child’s data with all recipients and for all purposes mentioned except in cases involving litigation related to unsafe medical products. In contrast, bereaved parents (T1B) did not express opposition to sharing data for this purpose. One bereaved parent indicated they would be very unlikely to share data for the purpose of performing research on health problems that might affect their family or child.

At all time points, most parents perceived that the potential benefits of data sharing outweighed the potential negative consequences (86–93%) (Figure S1). Amongst parents who responded at both T0 and T2 (*n* = 14), seven parents’ perceptions remained the same, four parents perceived data sharing more positively over time, and three parents perceived data sharing more negatively—although for two parents this was a positive to neutral change. Amongst parents who responded at both T0 and T1B (*n* = 8), six parents’ perceptions remained unchanged, and two parents perceived data sharing more negatively (Figure S2).

Table 3 summarizes the themes and quotes representing parents’ attitudes toward sending and testing their child’s sample interstate and overseas. We identified three themes: parents’ support and motivations for sharing; parents’ preferences for sharing; and parents’ attitudes toward privacy protection. All parents agreed to their child’s tissue samples being sent interstate and overseas for PRISM analysis. Their support for sharing was motivated by an anticipated benefit for their child or other children with cancer, research advancement, and positive experiences with data sharing. One parent expressed concern over the potential for medical insurance discrimination if their child’s genetic data was exposed, but still believed the outcomes of sharing outweighed any concerns (Parent 12179).

### 3.3. Research Question 2: Perspectives on Additional Tumor Biopsy Requests

Table 4 summarizes the themes and quotes representing parents’ and oncologists’ attitudes toward tumor biopsies. Parents (*n* = 5) whose child had undergone an additional biopsy for PRISM shared that they felt comfortable with the procedure as they believed it was necessary to achieve the best outcome for their child. Amongst parents whose child did not (*n* = 36) or parents who were unsure (*n* = 3; due to child undergoing numerous procedures) if their child required an additional biopsy, some expressed hesitancy when asked to consider the possibility of another operation. When deciding whether to pursue an additional biopsy, parents described weighing up the risks and benefits, as well as considering their child’s and experts’ opinions. Nine parents reported that their child had to provide extra blood samples for participation in the trial. All parents expressed comfort with this and praised the PRISM team for incorporating the blood collection into routine blood draws, as it reduced burden and discomfort on their family. *‘It didn’t seem like a particularly disruptive involvement on our part; it was just something that was a part of his care’* (Parent 11147). Several parents noted that their child was accustomed to blood sampling as part of their oncological care, particularly when a central line was in place, and therefore, it resulted in minimal impact. However, one parent shared that their child *‘wasn’t particularly happy’* (Parent 11006) about the additional blood collection but attributed this to a general sense of distress associated with attending the clinic. Another parent stated they would have been more hesitant to allow for PRISM samples if a separate needle procedure had been required.

### 3.4. Research Question 3: Perspectives on Confidentiality

Table 5 summarizes the themes and quotes representing parents’ and scientists’ attitudes toward confidentiality within the trial. We identified three themes from parent interviews: support for identification of the child’s sample, influence of family preferences on privacy, and confidence in scientists’ professionalism. Parents in our sample were either ambivalent or supportive of scientists seeing their child’s name on samples. Parents believed linking a name to the sample humanizes the data and gives recognition to their child as *“more than a number”* (Parent 12238). Parents also felt comfortable with scientists seeing their child’s name, as they felt it would highlight the significance of the sample being worked on. This perspective contrasts with the conventional view of samples as anonymized data and raises questions about whether, in some circumstances, identification may be beneficial. Parents’ privacy preferences tended to align with family characteristics. For example, the tendency to be more outspoken or more private about their child’s cancer. Additionally, some parents were amenable to the use of their child’s name, provided their child was also in agreement. Parents also expressed confidence in the professionalism of scientists, trusting them to uphold confidentiality requirements

## 4. Discussion

This is the first study to explore key stakeholders’ attitudes toward data sharing, additional biopsy requests, and patient confidentiality in a precision medicine trial for poor-prognosis childhood cancer.

Parents’ positive attitudes toward sharing their child’s clinical data aligned with previous research [30,31] and revealed parents were driven by altruism and a desire to advance research. These motivations were also consistent with parents’ initial reasons for participating in the PRISM trial [43]. Our results showed that most parents’ views remained stable over time, even after receiving trial results or experiencing bereavement, suggesting parents’ support was not contingent on treatment outcomes. Previously reported data sharing concerns, such as potential discrimination by insurance companies [44,45,46,47,48,49,50,51,52], also emerged in our study. In September 2024, Australia introduced a legislative ban on such discrimination [53], which may help address long-term concerns; however, uncertainty about its implementation persists [54]. These findings highlight the need for clear, up-to-date information resources for enrolling families.

Parents showed less support towards sharing data for use in medical lawsuits, possibly reflecting discomfort with sharing data for commercial purposes [49,51,52]. Parents were most willing to share data with scientists from universities and non-profits, but also expressed support for sharing with medical companies. This may reflect recognition of their role in drug development [44] and the need for therapeutic research [46] to address the lack of targeted therapy for children, which is often a barrier in pediatric precision medicine trials [55,56].

All parents agreed to send their child’s tissue samples interstate and overseas for analysis. One possible reason for such high acceptance could be that parents were interviewed after they had received their child’s precision medicine trial results, and therefore perceived tissue sharing as having benefited their child and favored research advancement [57,58,59,60,61]. Only one parent expressed concern regarding potential insurance discrimination in the case of a privacy breach. Parents’ general lack of concern could be attributed to a sense of trust. In another study of pediatric data sharing, parents’ lack of privacy concerns reflected their trust in the primary researcher, with this trust extending to any decisions that the primary researcher made [62]. Response bias may also be a factor as parents who chose to participate in PRISM-Impact may have held more favorable views about research compared to the broader population of families undergoing precision medicine.

Overall, parents and oncologists supported additional biopsies, prioritizing safety, informed consent and potential patient or research benefit. Considering parents often defer complex decisions to oncologists [63,64], clinicians play a key role in ensuring families are informed and their expectations are managed [17,65]. Oncologists in our study also weighed population-level benefits (i.e., research value) in requesting biopsies for molecular profiling in children [66]. These findings underscore the need to balance research value with ethical principles, including beneficence, non-maleficence and respect for the child’s assent/dissent, particularly in poor prognosis settings where equipoise must be considered.

Parents and scientists in our study provided novel insights into patient confidentiality in a precision medicine trial. Parents and scientists value identifiable information in humanizing data. Parents appreciated their child being acknowledged as ‘more than a number’, reflecting a growing sentiment that patients want to be treated as people and a belief that they will receive better care if healthcare professionals know more about them [67]. While scientists felt a stronger emotional connection at the research clinical interface [17,33]. Precision medicine trials encourage closer collaboration between patient-facing and non-patient-facing professionals [33]. Though traditionally working with de-identified data, research scientists in precision medicine trials are increasingly exposed to individual cases, particularly during MTB meetings and show heightened care in managing confidentiality.

### Strengths and Limitations

A key strength of our study was the inclusion of multiple stakeholders in a national trial, allowing us to capture diverse views, including those of bereaved parents. While the absolute number of bereaved parents was small, their participation rate was high. Our large parent sample at enrolment (T0) was also a strength, though attrition over time was likely due to health deterioration or escalating demands of care for their child, thus limiting our ability to assess changes in perceptions over time. This sample was likely not representative of all families undergoing PM; those who opted to participate may have had particularly positive or negative experiences. We were unable to explore reasons for non-participation due to ethical restrictions. Future studies should examine families’ reasons for declining participation to explore further. Not all domains of interest were explored using a mixed method approach, i.e., confidentiality and attitudes towards biopsies were assessed solely through interviews (T1). Lastly, our study was limited by the exclusion of non-English-speaking parents. Given that culturally and linguistically diverse participants could have additional concerns regarding trial procedures or informed consent [68], further insight into these participants’ perspectives is necessary.

## 5. Conclusions

This study demonstrates that parents, oncologists, and research scientists show high levels of acceptance towards key procedural aspects of precision medicine trials, including additional biopsies, data sharing, and patient confidentiality, which, for the most part, outweigh the concerns expressed. Parents enduring support for data sharing, even after bereavement, suggest that their motivations extend beyond a hope for therapeutic outcomes from the trial for the benefit of their child. Parents’ willingness to accept additional biopsy procedures highlights the importance of informed consent and careful management of expectations in precision medicine trials. For oncologists, the findings point to an ongoing ethical responsibility to balance patient risk–benefit with research value, while scientists reflected on their responsibilities of working with identifiable patient information and maintaining confidentiality.

## Figures and Tables

**Table 1:** Demographics of children of participating parents in PRISM-Impact.

Characteristics of Children Whose Parents Participated in the Study Enrolled (*n* = 110)	
Age at T0, years	
Mean (SD)	8.98 (5.6)
Range	0–17
(Missing)	-
Age at diagnosis, years	
Mean (SD)	7.81 (5.5)
Range	0–17
(Missing)	1
Gender, n (%)	
Female	55 (50)
Male	55 (50)
Diagnosis, n (%)	
Central nervous system tumor	40 (36.4)
Sarcoma	35 (31.8)
Leukemia/Lymphoma	18 (16.4)
Neuroblastoma	8 (7.3)
Other	9 (8.2)
Relapse prior to PRISM consent, n (%)	
Yes	56 (50.9)
No	54 (49.1)

Note: SD = standard deviation, *n* = number.

**Table 2:** Demographics of scientists and oncologists.

	Scientists (*n* = 10)	Oncologists (*n* = 26)
Site, *n* (%)		
Sydney Children’s Hospital	-	10 (38.5)
Royal Children’s Hospital, Melbourne	-	2 (7.7)
Perth Children’s Hospital	-	6 (23.1)
Queensland Children’s Hospital	-	2 (7.7)
John Hunter Children’s Hospital, Newcastle	-	3 (11.5)
Women’s and Children’s Hospital, Adelaide	-	3 (11.5)
Gender, *n* (%)		
Female	4 (40)	12 (46.2)
No. of years of practice, mean (*SD*), range *	8.52 (10), 1–30	-
No. of years working in pediatric oncology, mean (*SD*), range *	-	15.61 (10.9), 6–50
Percentage of time dedicated towards research, mean (*SD*), range	100 (100), 100–100	35.4 (27.7), 5–100

* Item not administered to both groups, *SD* = standard deviation; *n* = number.

**Table 3:** Themes and quotes representing parents’ attitudes toward their child’s tissue sample being sent and tested overseas and interstate.

Support and Motivations for Sharing
Anticipated benefits
“I’m extremely comfortable if we can help, you know obviously my first priority is [my child] and if we can get a cure for him that’d be amazing, but if we can help other people, other children along the way that have been diagnosed with the same awful disease. If his tissue is going to help, then yeah, I’m more than happy to have [it shared].” (Parent 21267)
Research advancement
“I feel a bit I don’t know relieved really that it’s all going towards getting more answers you know. Maybe even not for us but for the type of cancer [my child] has…” (Parent 51383)
Preferences for sharing
Collaborative research
“There are so many advances made, and I don’t think any one country is particularly any better than any other. I think if there’s more people looking at a similar thing from different aspects then there’s more chance when they get together there would be a better outcome.” (Parent 51237)
“I’d rather [the sample] go to the place where they’ve got the best facilities to do [the analysis].” (Parent 11370)
Being informed
“I probably would like some feedback on the testing that’s been done and what they’ve found.” (Parent 51383)
Attitudes toward privacy protection
Confidence in privacy policies “I trust that the study and all the ethics surrounding it and the way the information is handled is done so in a way that is safe. You know everyone’s privacy concerns are taken care of.” (Parent 12238)
Concerns about privacy breach
“I suppose one of the concerns would be if [my child’s] genetic makeup and hence ours ends up in the hands of medical insurers and that affects insurance for [my child] in her future, or for us. That’s a concern.” (Parent 12179)

**Table 4:** Themes and quotes representing parents’ and oncologists’ attitudes toward additional tumor biopsies.

Parents
Experience with Additional Tumor Biopsies
Additional biopsy required
“We wanted to find out a little bit more [about our child’s cancer] and you know [a biopsy was] the only way to find out” (Parent 62414) “[Oncologist] assured us it [the biopsy] was for [the oncologists] research to try and find something for [Child’s Name] and it would help [oncologist] studies as well…I think families are just desperate at the time that they’ll jump on anything. Anything that’s new or improved. I’m definitely happy to pursue something like that, for sure” (Parent 12290)
Additional biopsy not required
“I think it was easier if it’s part of what’s already happening to the kids because there’s so much happening to them… I don’t know if I would have done it if it was an extra anesthetic, another invasive [procedure].” (Parent 51345) “They got tissue from when they did the debulking…it didn’t seem like a particularly disruptive involvement on our part; it was just something that was a part of his care…it was just part of the routine as far as we were concerned” (Parent 11147)
**Considerations when deciding whether to pursue an additional biopsy**
*Risk–benefit analysis*
Minimizing harm versus pursuing benefits “If he was already going through a lot of procedures, which he has, and we had to do an extra one to participate in the study, then I probably—I wouldn’t push him to do it. But if it was sort of, non-invasive or additional to something that was of negligible impact to him, then that would be fine” (Parent 31156)
“We didn’t want any specific procedures just for the study. [My child] was going through enough and that was really the feelings—you know—if it can help him even in the minutest change, we’ll take it. But it wasn’t going to be at the cost of an additional procedure, but we also recognize that the samples could help future patients, future studies so absolutely.” (Parent 42326)
Tumour-dependent
“Anything invasive, you know, which would include a surgery, then I’m a bit iffy about because where his inoperable tumor is, it’s quite dangerous. That’s why we’re not doing surgery.” (Parent 21260)
Child’s opinion
“I was fine as long as [my child] was happy with it.” (Parent 71280)
Expert opinion
“…but if [the biopsy is] what the surgeon recommends and says well–then you have to listen I suppose, they’re the experts.” (Parent 62259)
**Oncologists**
**Experiences with patients needing additional biopsies**
Parent accepting
“I think they [the parents] felt they’ve done their best for their child, and I think that was gratifying for them.” (Oncologist 017) “Not come across any negative connotations around it at all.” (Oncologist 021)
Parents not accepting
“We have been in a situation where parents did not want to, or the patient, didn’t want to put it further with a biopsy, where it was proposed to have a biopsy for PRISM. And then they say no if you think it’s unlikely to change anything, to change the outcome, we want to prioritize [the child’s] quality of life…’” (Oncologist 027)
**Impression of parents’ considerations**
Child’s safety
“…as long as it can be performed without any additional risks…for most cases I think the families are quite okay with the concept. But I guess, if getting additional material for the study involved any additional risks, then that’s a whole different” (Oncologist 006)
Being informed
“…if you explain to the family that this is what is done and there are no other ways of doing it and we are doing it within safety permits, most of them are okay with it.” (Oncologist 010)
Altruism
“Oncology families, they are more than happy to contribute to future data and to help future families because they know that what they are getting now is because of studies that have been done on previous patients’ samples.” (Oncologist 014)
Hope
“I haven’t experienced anyone who doesn’t want to participate when it’s available. If anything, they [parents] often want more, like a repeat biopsy or repeat analysis. (Oncologist 016)
**Favorable risk–benefit profile**
“I think, we do take safety at the forefront to make sure this actually a safe procedure. Of course, you acknowledge there is always risk. But you know, you feel that, you know, the benefit can outweigh the risk. (Oncologist 010)
“I think all these things [biopsies] carry risks and its risk–benefit. I think if you’re doing something anyway, there is no problem. Even if you’re taking a bit more of a sample. I don’t think that is a problem at all. I think the risk is doing a procedure that you would not otherwise do…it was the ethical risk.” (Oncologist 009)
**Informed consent**
“I think it’s important to discuss with the families and offer it as saying there is opportunity to try and identify unique, targetable treatments, but I think doing it with the realistic outcomes that we may more than likely not find anything…Obviously, being fully informed is a good way for families to then make a decision about whether they want to face that biopsy option or not.” (Oncologist 054) “I think that the family need to be, you know, have full informed consent, that point of view, as well as understand exactly what PRISM is about. Now that’s not to say it’s not worthwhile and, provided those things are carried out, then it’s not unreasonable to do that [additional biopsy]. (Oncologist 009)
**Research value**
“I think [re-biopsies] are going to become more and more standard practice mainly because of literature surrounding the change in the mutational landscape of tumors from the time of presentation to the relapse…” (Oncologist 054)

**Table 5:** Themes and quotes representing parents’ and scientists’ attitudes toward confidentiality.

Parents
Support for the identification of the child’s sample
Having a name humanizes the data
“I mean at the end of the day there’s a face attached to [the sample] and that’s the reason we’re all doing this you know…because there’s people that are loved ones and it’s not just a piece of tissue in a jar, it’s someone’s life at the end of the day…it’s more than a number so I don’t really have an issue I think it drives home the importance of the work that they’re doing essentially.” (Parent 12238)
“I actually feel for them [the scientists] because it actually makes it more personal putting a name instead of just a sample. Personally, I don’t have a problem with it, but it does give me empathy on their side, and it may actually be harder for them” (Parent 22018)
Influence of personal experiences
“It’s easier to identify samples by name rather than by number. I mean, researchers have got to do their research. I’ve been involved in doing Honours research myself when I was at uni, so I know what it’s like to be on the other side of it.” (Parent 11147)
“You can take a whole heap of physiologies and do your tests on that and that will give you a certain amount of information. If you were actually to treat it [cancer] holistically you would take that information and you would also take the story and the history” (Parent 11370)
Being hopeful of positive outcomes
“I don’t have a problem with that [child’s name being seen] at the end of the day I look at it as testing to be able to assist, yeah, it’s not testing for a negative outcome, it’s something for a positive. So, for me that makes me feel okay with it.” (Parent 71386)
**Influence of family preferences on privacy**
Outspoken
“It doesn’t bother me awfully, except that we’re a reasonably outspoken family anyway about [my child’s] journey to get research and funding and support, so I mean it wouldn’t be hard to join two and two together…I’ve been on the television and supported the [charity name] to get money for their sarcoma researchers can do research for other kids that have secondary cancers like my son did.” (Parent 51284)
Private
“I suppose as long as [the scientists] are subject to confidentiality requirements because we’re definitely a very private family.” (Parent 77106)
Child-oriented
“We’ve talked to [my child] about that [scientists seeing child’s name] and she said the more people that know, the better.” (Parent 42151)
“…as long as [my child] was right with it and he was asked everything that was, I always asked him if everything was okay in doing it so, yeah.” (Parent 71280)
**Confidence in scientists’ professionalism**
“I’m confident–I did read the privacy information very carefully when the initial paperwork was given out, and I was confident that the way it was being handled was appropriate for the type of work they were doing.” (Parent 11147)
“Oh look, it’s not a problem. I feel they [scientists] must have professional ethics and guidelines and things like that, I don’t really have a problem if his name was on it or not, if he’s just a number, I always think that must be their professional job.” (Parent 71182)
**Scientists**
**Understanding confidentiality requirements** Completed privacy training “…we all have to go through a training in privacy before we even begin work, so yes, I think everyone in the trial is very aware of how important it is to keep like–we are even careful to not leave labels of stuff lying around, make sure there are no confidential emails just up on your screen when you are not at your desk, so yes, we are careful about it…” (Scientist 065)
**Experience of maintaining confidentiality** Role-dependent access “I think it’s all fairly confidential–only limited persons know the actual identity in terms of the name and date of birth, but when we process the samples, they’re all coded, like de-identified, and we are working with the patient’s ID.” (Scientist 040) “Clinicians will use the patient’s name, but I don’t think I’ve ever operated using the patient’s name. Always with our samples they’re deidentified at tumor bank when they’re received and then from that point onwards it’s always P number, by material IDs, very rarely names.” (Scientist 064) “You do need to have [patient names] because we’re writing reports as well as the presentations” (Scientist 063) *Challenges maintaining confidentiality* “It’s all well and good if you talk to your colleagues in the lab, you talk about numbers and deidentified numbers, but when you talk to the clinicians, so you talk to the oncologist, you talk to the pathologist, they like to use names, but then in the [shared] office area, it’s a little bit confined, I find it’s really hard to try to say ‘I can’t talk about patient names’, when you’ve got a clinician who asks ‘what’s going on with this patient?’” (Scientist 031)
**Attitudes toward seeing patient names** *Patient names humanize the data* “[PRISM samples] are a bit different because we are allowed to see patient names. We try not to, but identifiability is built into the PRISM study. At some point we write a report with the patient’s name on it… I think we’re used to it now. I remember it being confronting, but I feel like that’s part of essentially doing a pathology service which—which we are doing for PRISM. (Scientist 043) “It’s necessary [to see the names] because it involves clinical care. It helps humanize the data that we’re looking at…we know each data point represents a patient and [the patients] are relying on the outcome.” (Scientist 043) “[Seeing patient names] really drives home the fact that it is a child and not just a number, it really does. And sometimes we are even made aware of the age, and that kind of, you know, it can sometimes break your heart a little bit, when it’s like a two-year-old or a three-year-old.” (Scientist 065)

## Data Availability

The data presented in this study are available on request from the corresponding author. The data are not publicly available due to privacy/ethical reasons.

## Supplementary Materials

The following supporting information can be downloaded at: https://www.mdpi.com/article/10.3390/jpm15110531/s1, Supplementary note: Factors associated with participation and attrition (parents); Table S1. Questionnaire and semi-structured interview questions; Table S2. Baseline characteristics of participating parents; Figure S1. Parents’ perceptions of the benefits and negatives of data sharing; Figure S2. Parents perceptions toward potential benefits of sharing anonymous, individual clinical trial data weighed against the potential negative consequences.

## Abbreviations

The following abbreviations are used in this manuscript:

PRISM	PRecISion Medicine for Children with Cancer
MTB	Molecular Tumor Board
ZERO	Zero Childhood Cancer National Precision Medicine Program
