# Supplementary material for: Data Sharing, Biopsies and Patient Confidentiality in a Precision Medicine Trial for Childhood Cancer: A Mixed Method Study of Parents, Oncologists, and Scientists’ Perspectives
Source: J Pers Med. 2025 Nov 2;15(11):531. doi: 10.3390/jpm15110531 (PMC12653184; doi:10.3390/jpm15110531)
Supplement: Supplementary file 1 [file jpm-15-00531-s001.zip › jpm-3828293-supplementary.pdf]

**Supplemental note: factors associated with participation and attrition (parents)**

We examined characteristics associated with parents' decision to participate in PRISM-Impact using a logistic regression model. The outcome variable was participation defined as return of the T0 questionnaire, and the covariates were patient age, treating hospital, initial diagnosis, and relapse status. This model found strong evidence that after adjusting to patient age, treating hospital, and initial diagnosis, parents whose child had relapsed prior to PRISM enrolment had a higher participation rate than parents whose child had not relapsed (OR 2.46, 95% CI 1.53-3.96, p=< 0.001).

We examined characteristics associated with parents maintaining participation in PRISM-Impact at T2 or T1B using logistic regression models. The dependent variable was return of the T2 or T1B questionnaire respectively, and the covariates for both models were the child's relapse status and whether the family had received treatment recommendations. Parents who were bereaved and who were still being followed up were excluded from analysis at T2, and parents who had not reached six months post-bereavement were excluded from analysis at T1B. At both T2 (child prior relapse OR = 0.56, 95% CI 0.22 – 1.42, p = 0.224; treatment recommendations OR = 0.49, 95% CI 0.18-1.31, p=0.157) and T1B (child prior relapse OR = 1.39, 95% CI 0.44-4.38, p = 0.574; treatment recommendations OR = 0.69, 95% CI 0.20- 2.31, p=0.544), we found no strong evidence that maintaining participation differed according to either covariate modelled.

**TABLE S1** Questionnaire and semi structured interview questions

| Questionnaires | | | | | |
|---|---|---|---|---|---|
| **Aim** | **Item** | **Measure and response option** | **T0** | **T2** | **T1B** |
| Demographics | • Age<br>• Gender<br>• Cultural background<br>• Highest level of education<br>• Current employment status<br>• Household income<br>• Rurality (postcode)<br>• Marital status<br>• Number of other children | Combination of open ended (e.g. postcode) and forced choice options (e.g. level of education) depending on the question. | **X** | | |
| Thoughts on data sharing | • How likely would you be to allow your child's anonymous, individual clinical trial data to be shared with… *(Adapted from Mello et al [1])*<br>- Scientists in universities and other not for profit organizations?<br>- Scientists in companies developing medical products, such as prescription drugs? | "Very likely"<br>"Somewhat likely"<br>"Neither likely nor unlikely"<br>Somewhat unlikely"<br>"Very unlikely" | **X** | **X** | **X** |
| | • How likely would you be to allow your child's anonymous, individual clinical trial data to be used in the following ways? *(Adapted from Mello et al [1])*<br>- To help scientists check the accuracy of research results announced by other scientists or companies (by re-doing the analyses)?<br>- To help patients and groups of patients learn more about health problems that affect them.<br>- To do research on health problems that affect my family or my child.<br>- To help get answers to scientific questions faster using information that others have already gathered.<br>- To do research that will help others.<br>- To help lawyers prove their case in lawsuits claiming that medical products are unsafe.<br>- To learn more about diseases that only a small number of people have (by combining data from many clinical trials). | "Very likely"<br>"Somewhat likely"<br>"Neither likely nor unlikely"<br>Somewhat unlikely"<br>"Very unlikely" | **X** | **X** | **X** |

| | | | | | |
|---|---|---|---|---|---|
| | • Overall, how do you think the potential benefits of sharing anonymous, individual clinical trial data weigh against the potential negative consequences? (Check one) *(Adapted from Marron et al [2]* | “Negatives strongly outweigh the benefits”<br>“Negatives moderately outweigh the benefits”<br>“Negatives outweigh the benefits a little”<br>“Benefits and negatives are equal”<br>“Benefits outweigh the negatives a little”<br>“Benefits moderately outweigh the negatives”<br>“Benefits strongly outweigh the negatives” | **X** | **X** | **X** |
| | - | | | | |

**Semi-structured interview questions (T1)**

| **Cohort** | **Item** |
|---|---|
| Parents | • Did your child need to have any extra, or more invasive, procedures or tests before participating in the PRISM study? *(adapted from Cohen et al.[3]).*<br>*If yes:*<br>- What was the procedure?<br>- How comfortable were you with your child having these extra procedures?<br>- Do you think it is ethical to offer extra tests and procedures like this to families?<br>• How comfortable do you feel that part of the samples are sent and tested overseas and interstate?<br>• How do you feel about that scientists who do the testing are able to see your child’s name? |
| Oncologists | • Demographics:<br>- Age<br>- Gender<br>- Professional title<br>- Number of years of practice?<br>- Number of years working in paediatric oncology?<br>- What percentage of your time is dedicated towards research? |

| | |
|---|---|
| | • Conducting new research testing such as that offered by PRISM requires a change in usual practice, for example taking a new biopsy at relapse or taking a larger biopsy. What is your impression of parents' willingness to do this in a safe manner, particularly when there is no guarantee that it will benefit their child? Do you think this is appropriate?<br>• Were any of your patients asked to provide an extra, or larger than normal, biopsy this year?<br>*If yes:*<br>- How did they respond?<br>- What were your thoughts at this time for them?<br>• Do you feel it is appropriate to offer families larger, or additional biopsies, as part of PRISM? Why/why not? |
| Scientists | • Demographics:<br>- Age<br>- Gender<br>- Professional title<br>- Number of years of practice?<br>- Number of years working in paediatric oncology?<br>- What percentage of your time is dedicated towards research?<br>• Do you understand the confidentiality of the samples you deal with? Probe: How do you find it dealing with the confidentiality of patient samples? (Probe hard/easy) What is it like seeing the patient's name on the samples? |

[1] Mello MM, Lieou V, Goodman SN. Clinical Trial Participants' Views of the Risks and Benefits of Data Sharing. New England Journal of Medicine. 2018;378(23):2202-11; [2] Marron JM, DuBois SG, Bender JG, Kim A, Crompton BD, Meyer SC, et al. Patient/parent perspectives on genomic tumor profiling of pediatric solid tumors: the Individualized Cancer Therapy (iCat) experience. Pediatric Blood Cancer. 2016; 00:1–9. doi: 10.1002/pbc.26137.; [3] Cohen B, Roth M, Marron JM, Gray SW, Geller DS, Hoang B, et al. Pediatric Oncology Provider Views on Performing a Biopsy of Solid Tumors in Children with Relapsed or Refractory Disease for the Purpose of Genomic Profiling. Ann Surg Oncol. 2016;23(Suppl 5):990-7.

**Table S2. Baseline characteristics of participating parents.**

| | Parents who completed data sharing questions. (n=126) | Parents who completed an interview. (n=53) |
|---|---|---|
| **Age at T0, years** | | |
| Mean (SD) | 42.89 (7.4) | 43.3 (7.3) |
| Range | 22-67 | 29-67 |
| (Missing) | 4 | 3 |
| **Gender, n (%)** | | |
| Female | 77 (61.1) | 37 (69.8) |
| Male | 49 (38.9) | 16 (30.2) |
| **Relapses prior to PRISM consent, n (%)** | | |
| Yes | - | - |
| No | - | - |
| **Cultural or language diversity, n (%)** | | |
| First language English, Western/European | - | 40 (75.4) |
| First language English, non-Western/European | - | 4 (7.5) |
| First language other than English | - | 8 (15) |
| (Missing) | - | 1 (1.8) |
| **Highest level of education, n (%)** | | |
| High school only | 18 (14.3) | 6 (11.3) |
| Post high school (inc. vocational training) | 108 (85.7) | 47 (88.7) |
| **Employment, n (%)** | | |
| Employed: Full-time | 62 (49.2) | 24 (45.3) |
| Employed: Part-time/casual | 28 (22.3) | 16 (30.2) |
| Employed: Did not specify | 2 (1.6) | 0 (0) |
| Not employed: Actively seeking work | 2 (1.6) | 1 (1.9) |
| Not employed: Not seeking work/retired/student | 10 (7.9) | 5 (9.4) |
| Not employed: Home duties | 21 (16.7) | 7 (13.2) |
| Not employed: Did not specify | 2 (1.6) | 0 (0) |
| **Household income, n (%)** | | |
| Nil income | 5 (4) | 2 (3.8) |
| Less than $29,999 | 5 (4) | 3 (5.7) |

| | | |
|---|---|---|
| $30,000-$59,000 | 12 (9.5) | 8 (15.1) |
| $60,000-$89,000 | 22 (17.5) | 9 (17) |
| $90,000-$120,000 | 18 (14.3) | 6 (11.3) |
| Greater than $120,000 | 47 (37.3) | 22 (41.5) |
| Prefer not to answer | 17 (13.5) | 2 (3.8) |
| (Missing) | - | 1 (1.9) |
| **Rurality, n (%)** | | |
| Capital city | 93 (73.8) | 37 (69.8) |
| Other metropolitan center | 13 (10.3) | 5 (9.4) |
| Rural/remote area | 19 (15.1) | 11 (20.8) |
| (Missing) | 1 (0.8) | - |
| **Marital status, n (%)** | | |
| Currently married / de facto | 108 (85.7) | 44 (83) |
| Separated / divorced / previous de facto/ widowed | 18 (14.3) | 9 (17) |
| **Number of other children, n (%)** | | |
| 0 | 24 (19) | 0 |
| 1 | 51 (40.5) | 12 (22.6) |
| 2-3 | 45 (35.7) | 33 (62.3) |
| 4+ | 5 (4) | 7 (13.2) |
| (Missing) | 1 (0.8) | 1 |

Note: SD = standard deviation, n = number.

**Figure S1: Parents' perceptions of the benefits and negatives of data sharing at T0 (n=106), T2 (n=29) and T1B (n=13)**

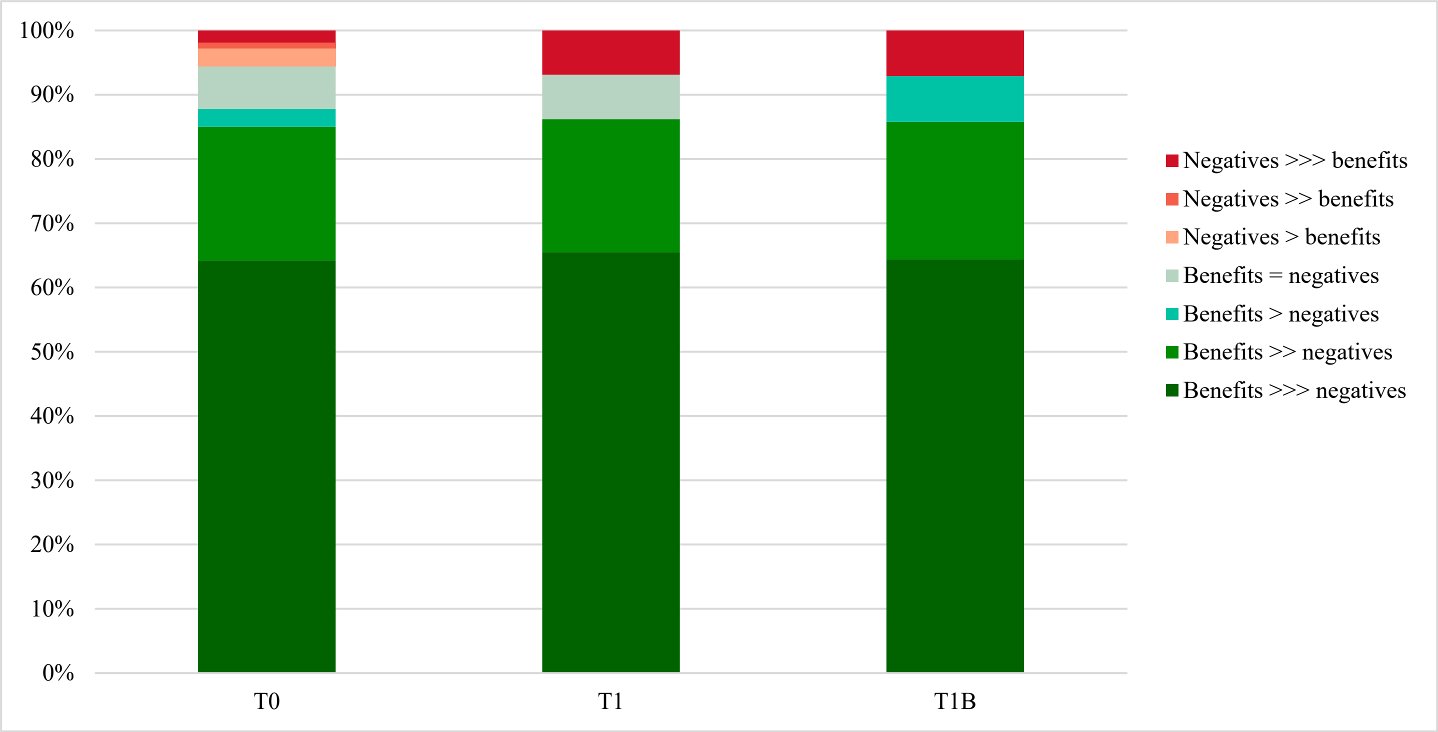


**Note:** >>> strongly outweigh, >> moderately outweigh, > slightly outweigh

**FIGURE S2: Parents' perceptions toward potential benefits of sharing anonymous, individual clinical trial data weighed against the potential negative consequences (a) from enrolment (T0) to one year post enrolment (T2), and (b) enrolment (T0) to post bereavement (T1B) amongst parents who responded at both timepoints.**

**a**

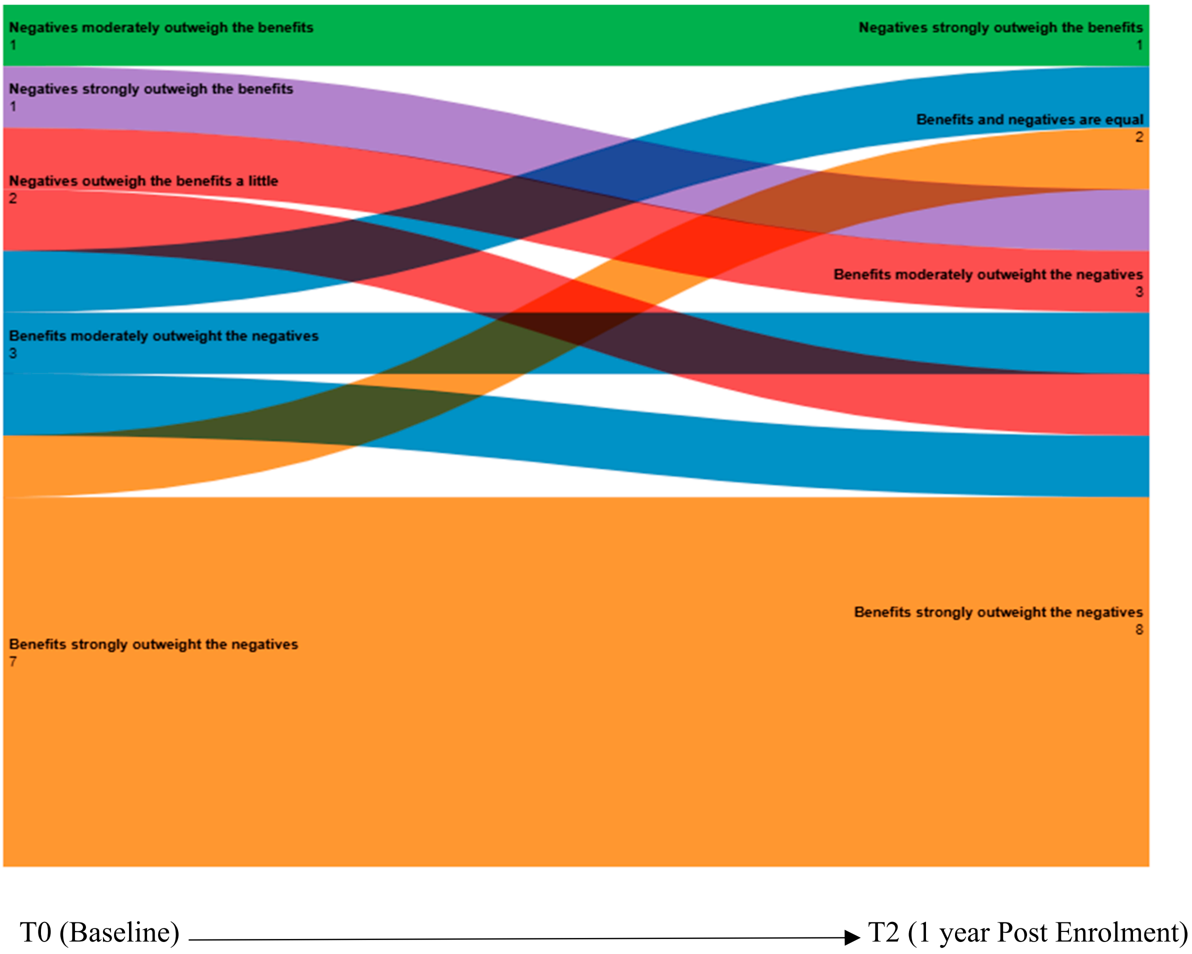

**b**

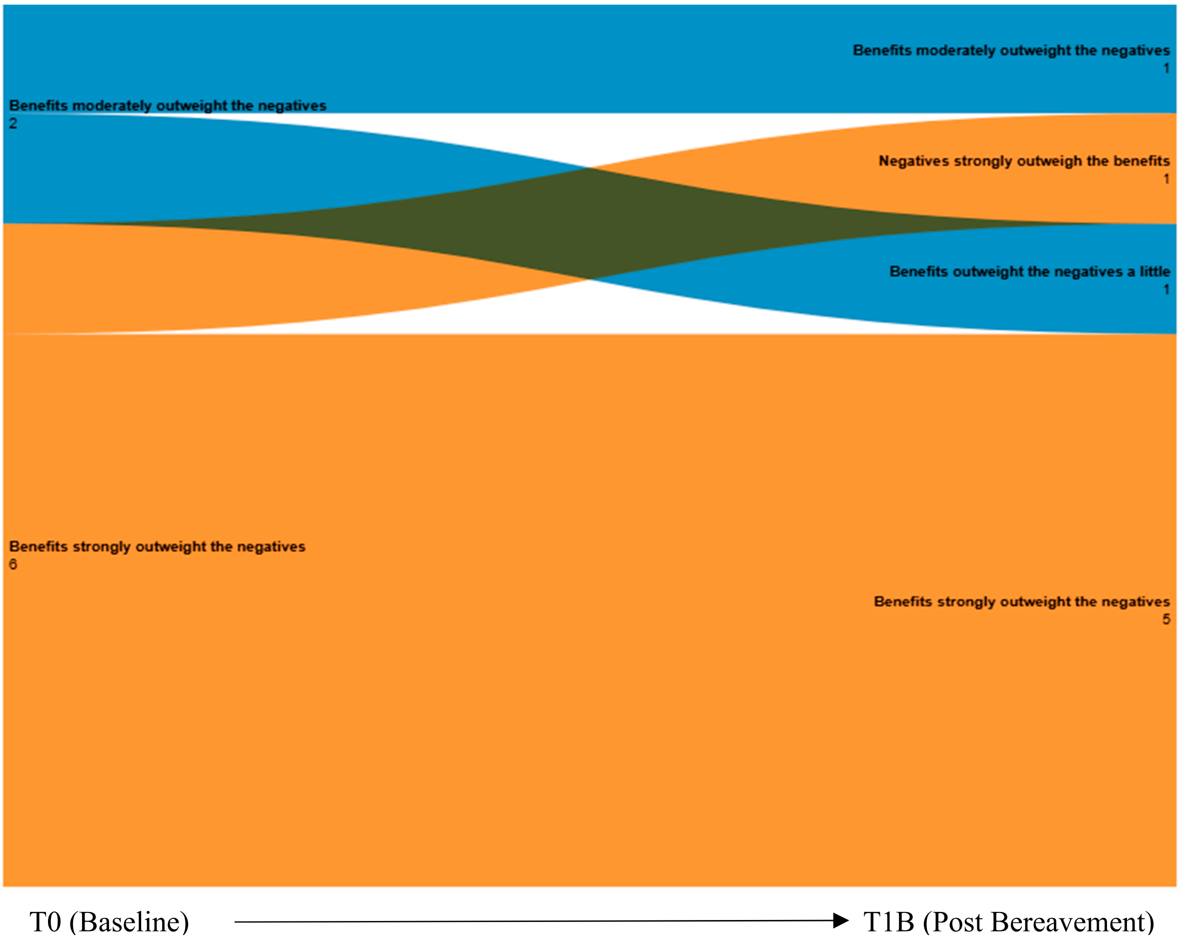


**Notes:** The alluvial plots in this figure visually represent the proportion of participants' responses sharing of data question at Time 0 and Time 1 and Time T1B, as well as any changes in participants' individual responses over time.
